# Retinal Stem/Progenitor Cells Derived From Adult Müller Glia for the Treatment of Retinal Degeneration

**DOI:** 10.3389/fcell.2021.749131

**Published:** 2021-09-29

**Authors:** Lay Khoon Too, Matthew P. Simunovic

**Affiliations:** ^1^Save Sight Institute, The University of Sydney, Sydney, NSW, Australia; ^2^Sydney Eye Hospital, Sydney, NSW, Australia

**Keywords:** Müller glia, retinal degeneration, stem-cell therapy, regeneration, reprogramming

## Abstract

Over the past two decades, progress in our understanding of glial function has been revolutionary. Within the retina, a subset of glial cells termed the “Müller glia (MG),” have been demonstrated to play key roles in retinal homeostasis, structure and metabolism. Additionally, MG have also been shown to possess the regenerative capacity that varies across species. In teleost fish, MG respond to injury by reprogramming into stem-like cells capable of regenerating lost tissue. The expression of stem/progenitor cell markers has been demonstrated broadly in mammalian MG, including human MG, but their *in vivo* regenerative capacity appears evolutionarily limited. Advances in stem cell therapy have progressively elucidated critical mechanisms underlying innate MG reprogramming in teleost fish, which have shown promising results when applied to rodents. Furthermore, when cultured *ex vivo*, MG from mammals can differentiate into several retina cell types. In this review, we will explore the reparative and regenerative potential of MG in cellular therapy approaches, and outline our current understanding of embryonic retinal development, the stem-cell potential of MG in adult vertebrate retina (including human), and microenvironmental cues that guide MG reprogramming.

## Introduction

Retinal degenerative disease is the leading cause of irreversible blindness. Inherited retinal disease (IRD), affecting 1 in 2,000–3,000 individuals, is the commonest cause of vision loss in the working-age populace (Liew et al., 2014), whilst age-related macular degeneration (AMD) is a leading cause of vision loss in those over 50 years (Blindness and Vision Impairment Collaborators, and Vision Loss Expert Group of the Global Burden of Disease Study, 2021). There is a significant degree of genetic diversity in IRD, with more than 300 genes implicated to date: testing in specialized clinics can elucidate the genotype in only about 70% of cases (Moore, 2017). AMD, on the other hand, is multifactorial, with significant genetic and environmental contributions.

Over the past two decades, there have been revolutionary advances in both IRD and AMD management. For example, anti-vascular endothelial growth factor agents have improved long-term outcomes following the onset of neovascular AMD. Gene therapy offers promise to those with IRD. While the FDA approval of the first gene therapy (Luxturna^TM^) in 2017 provides hope for patients with biallelic RPE65mutation-associated retinal dystrophy (Nature Biotechnology, 2018), this represents less than 1% of patients with retinal dystrophies (Chao et al., 1993; Apte, 2018). Furthermore, such gene-specific therapy may not be useful for patients with end-stage retinal disease due to irreversible retinal damage.

The genetic and phenotypic heterogeneity of retinal degenerative disease has led to the development of gene- and pathway-agnostic therapeutic approaches. These include cellular therapy (reviews in, Mead et al., 2015; Singh et al., 2020; Wang et al., 2020; West et al., 2020), electronic retinal implants (Mills et al., 2017), and optogenetics (reviews in, Dalkara et al., 2015; Duebel et al., 2015; Chaffiol and Duebel, 2018; Fortuny and Flannery, 2018; Simunovic et al., 2019). Stem cell therapy involves delivering donor cells to replace lost neuronal cells, or to prevent further degeneration of existing host neurones. Electronic retinal implants produce a visual percept through electrical stimulation of surviving neurones and may be epiretinal, subretinal, or suprachoroidal. Optogenetic approaches involve introducing light-sensitive proteins via gene therapy to confer light sensitivity to remaining secondary/tertiary retinal neurones, which are naturally not light-sensitive (effectively converting them into photoreceptors, i.e., light-sensitive primary neurones). While optogenetic approaches are only just undergoing translation (Sahel et al., 2021), multiple phase I/II clinical trials of stem cell transplantation have largely supported the safety of stem cell therapy in humans, with some signal of functional benefit in at least a proportion of patients (da Cruz et al., 2018; Wang et al., 2020). Although there has been a proliferation of clinics and treatment centers offering spurious treatments, sometimes with disastrous outcomes (Turner and Knoepfler, 2016; Kuriyan et al., 2017), advances in medical technology have enabled the production of clinical-grade cell-based therapies, and there remains great hope for cellular therapies to treat retinal degeneration (Sharma et al., 2019).

Various sources of stem and progenitor cells, including Müller glia (MG), fetal retinal progenitor cells, ciliary epithelium-derived stem cells, umbilical tissue-derived stem cells, bone marrow-derived mesenchymal stem cells, embryonic stem cells (ESCs) and induced pluripotent stem cells (iPSCs), have been studied for their potential to rescue retinal degeneration (Canto-Soler et al., 2016). The different types of stem and progenitor cells may rescue or restore vision via two broad mechanisms: (1) Replacement of lost cellular populations, e.g., photoreceptors or retinal pigment epithelium (Gonzalez-Cordero et al., 2017; Zhao et al., 2017; Ribeiro et al., 2021); (2) Neuroprotection through general immune-modulatory or neuroprotective effects, which may occur directly via material transfer, or indirectly in a paracrine fashion (Pearson et al., 2016; Singh et al., 2016; Nickerson et al., 2018). This review will focus on the therapeutic potential of MG and their derivatives.

## The Formation of Müller Glia During Retinal Development

**FIGURE 1:**
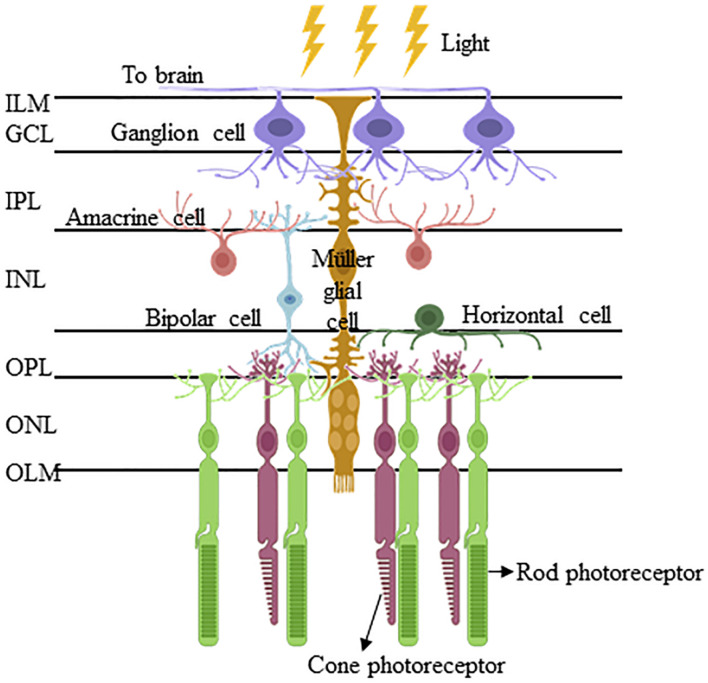
Müller glia and their interacting retinal cells are organized in a highly conserved manner in the vertebrate retina. The Müller glia are the only retinal cell type that span the entire retinal thickness and interact with all the six retinal cell types, namely retinal ganglion cells, amacrine cells, bipolar cells, horizontal cells, and rod/cone photoreceptors. Their cell bodies reside in the inner intermediate layer of the inner nuclear layer (INL), from which the Müller glial processes extend apically to form the inner limiting membrane (ILM) of the retina, and basally to delineate the outer limiting membrane (OLM). Between ILM and OLM, their processes interact with those of inner neurones (i.e., retinal ganglion cells, amacrine cells, and bipolar cells) and inner/outer neurones (i.e., bipolar cells, horizontal cells, and photoreceptors) to constitute the inner plexiform layer (IPL) and outer plexiform layer (OPL), respectively. (Abbreviations: ILM, inner limiting membrane; GCL, ganglion cell later; IPL, inner plexiform later; INL, inner nuclear later; OPL, outer plexiform later; ONL, outer nuclear layer; and OLM, outer limiting membrane).

The vertebrate retina is an embryonic derivative of the diencephalon of the forebrain. The retina and diencephalon share a common developmental program that is phylogenetically ancient, being conserved over 500 million years (Lamb et al., 2008). Early in embryogenesis, the eye field region in the diencephalon grows laterally into two optic vesicles, which subsequently invaginate to form the double-walled optic cup that, in turn, produces the neural retina and the retinal pigment epithelium (Bassett and Wallace, 2012; O’Hara-Wright and Gonzalez-Cordero, 2020). The vertebrate neural retina comprises seven major retinal cell types (six types of neurones and one type of glial cell) organized into five major lamellae, with three lamellae of cell bodies separated by two plexiform lamellae (Figure 1). All the major retinal cell types are generated from a pool of multipotent retinal progenitor cells in a highly conserved order, where retinal ganglion cells (RGCs) emerge first, followed by cones, horizontal cells and most of the amacrine cells at the early developmental phase, and bipolar neurones, rods and MG postnatally (Cepko et al., 1996). It should be noted that there is considerable overlap in the staging; however, the consensus is that RGCs are differentiated first, then rod photoreceptors and MG last (Centanin and Wittbrodt, 2014). Furthermore, individual progenitor cell line fates may be regulated reproducibly by lineage (Bassett and Wallace, 2012) or occur stochastically (Gomes et al., 2011). Interestingly, several morphological studies in the late 19th century on vertebrate retinas demonstrated early prenatal differentiation of MG, suggesting MG and retinal neuronal differentiation is spontaneous (Uga and Smelser, 1973; Bhattacharjee and Sanyal, 1975; Lemmon and Rieser, 1983; Prada et al., 1989). This is in contrast to the general belief that MG emerge last, according to birth-dating studies that use ^3^H-thymidine to mark terminally mitotic cells (Fujita and Horii, 1963; Hollyfield, 1972; Kahn, 1974; La Vail et al., 1991; Stiemke and Hollyfield, 1995; Rapaport et al., 2004). Further morphological studies suggest that the prenatally differentiating MG remain mitotically active, which explains why the early differentiating MG were not birth-dated by ^3^H-thymidine labeling (Hollyfield, 1968; Stiemke and Hollyfield, 1995). This is corroborated by the currently well-established perspectives that differentiated MG in the adult mammalian retina can proliferate *in vivo* under specific pathologic conditions and *in vitro* (Eastlake et al., 2021).

## Functions of Müller Glia in the Retina

Müller glia were first described by Heinrich Müller in the mid-19th century. They are specialized radial glial cells that in all vertebrate species have two processes which stem from their soma, located in the inner nuclear layer. These processes extend apically to approach the vitreous cortex where they form the internal limiting membrane, and basally to the subretinal space where they exhibit microvilli (Bringmann et al., 2006). With their unique architecture – that enables contact with all echelons of retinal neurones – MG serve several fundamental roles to support retinal homeostasis and maintain the inner blood-retinal barrier. These roles have been comprehensively reviewed elsewhere (Bringmann et al., 2006; Reichenbach and Bringmann, 2013), and include:

1. Regulation of the extracellular space composition (electrolyte and water homeostasis).
2. Modulation of synaptic activity of the inner retina via uptake and exchange of the neurotransmitters glutamate and γ-aminobutyric acid.
3. Generation of glutamine, which serves as a substrate for metabolic pathways and neurotransmitter precursors.
4. Generation of antioxidants (e.g., glutathione) to combat oxidative stress.
5. Support of photoreceptor viability by secretion of neurotrophic factors, phagocytosis of outer segment disks, and support of photoreceptor outer segment assembly.
6. Neuroprotection by disposal of carbon dioxide; hence, regulating extracellular pH for functional neuronal activity.
7. Regulation of the blood-retina barrier, blood flow, and neurovascular coupling.
8. Decreasing reflection at the vitreous/retinal interface/acting as light guides.
9. Regulation of mechanical homeostasis.

Additionally, Eastlake and colleagues have recently reported the secretion of extracellular vesicles by MG cultured *in vitro* (Eastlake et al., 2021). These extracellular vesicles carry RNAs coding for neurotrophic factors and microRNAs that regulate axonal/neuronal growth via the PI3K/Akt pathway, which suggest a neuroprotective role/potential of MG.

Apart from the aforementioned roles, a subset of MG in the mature retina harbor stem/progenitor cell characteristics/potential. These cells have been reported in the mature cadaveric human retina, where they were found predominantly in the retinal periphery (Mayer et al., 2005; Bhatia et al., 2009; Too et al., 2017) and in the epiretinal membranes of patients with proliferative retinopathies (Mayer et al., 2003; Johnsen et al., 2012). Recently, we identified retinal progenitor cells of MG lineage in surgical retinal explants excised from the mid-periphery of living donors undergoing rhegmatogenous retinal detachment repair (Too et al., 2021). Together, these cells serve as a potentially important homologous – or autologous (if derived from living donors) – source of stem/progenitor cells that warrant further investigation of their potential in regenerative medicine.

## Stem-Cell Characteristics of Müller Glia

Müller glia are not conventional stem cells *per se*, due to their differentiated phenotype. However, they have been reported to display stem-cell characteristics that vary by animal species, retinal status and topographical location. The periphery is thought to contain a richer “MG stem-cell” niche than the central retina (Fischer and Reh, 2000; Raymond et al., 2006; Martinez-Navarrete et al., 2008; Too et al., 2017). One possible explanation for the proposed gradient of “stemness” may be deduced from the ontogenetic patterns of retinal development, where gradients of differentiation appear from inner to outer layers (Mann, 1928) and from center to the periphery (Prada et al., 1991), approaching the peripheral margin of the retina in the last stage of neurogenesis. Alternatively, MG in the periphery may be more frequently exposed to the stimuli for de-differentiation: for example, peripheral retinal degenerative changes are often observed in patients who have ostensibly otherwise normal retinae. The MG in the far peripheral retina, highly express known stem/progenitor-cell markers nestin (Bhatia et al., 2009) and CD44 (Too et al., 2017). Several studies also demonstrate early differentiation of MG in postnatal retinae, where cells with morphological characteristics of MG – or cells labeled with MG-specific reporters – remain mitotically active and behave like retinal progenitors (Hollyfield, 1968; Robinson et al., 1985; Stiemke and Hollyfield, 1995), with lineage-tracing evidence confirming their role as precursors of rod progenitors (Bernardos et al., 2007; Stenkamp, 2011). These observations suggest MG are endogenous retinal stem/progenitor cells during and following retinogenesis.

Stem-cell niches in the adult retina were initially reported by several researchers who observed regeneration of retinal neurones and restoration of retinal circuitry following surgical removal of small retinal explants in fish (Lombardo, 1968; Hitchcock et al., 1992; Hitchcock and Cirenza, 1994; Cameron and Easter, 1995; Cameron and Carney, 2000; Faillace et al., 2002). Subsequent studies of neurotoxicity and phototoxicity confirmed that these insults similarly trigger robust proliferative/regenerative responses in the fish retina (Maier and Wolburg, 1979; Raymond et al., 1988; Vihtelic and Hyde, 2000; Vihtelic et al., 2006; Fimbel et al., 2007). Given that MG are the only retinal cell type that span the entire retinal thickness and contact all other retinal neurones via their processes, they are therefore arguably well-placed to respond to insults by triggering endogenous regenerative events (Goldman, 2014). The origin of retinal progenitor cells, however, remained elusive until several lineage studies clearly identified MG as precursors of regenerated neurones (Bernardos and Raymond, 2006; Fausett and Goldman, 2006; Bernardos et al., 2007; Fimbel et al., 2007; Ramachandran et al., 2010), and blocking of of MG cell division was demonstrated to inhibit injury-mediated retinal regeneration (Thummel et al., 2008).

Similarly, retinal injury triggers MG proliferation and their expression of retinal progenitor cell markers in other lower, as well as higher, vertebrates. These transformed MG differentiate into retinal neurones in Xenopus (Langhe et al., 2017), postnatal chickens (Fischer and Reh, 2001), mice (Karl et al., 2008), and adult rats (Ooto et al., 2004). Although the potential for endogenous regeneration is yet to be explored in the human retina, adult MG with stem-cell phenotypes have been persistently reported (Lawrence et al., 2007; Bhatia et al., 2009; Too et al., 2017) with their proliferative ability and multipotency shown *in vitro* (Giannelli et al., 2011; Jayaram et al., 2014; Eastlake et al., 2019). Expression profiling studies of vertebrate retina suggest that MG share molecular similarities with retinal progenitor cells (Roesch et al., 2008; Jadhav et al., 2009). This exquisitely differentiated, yet mitotically active, phenotype of MG has therefore received significant interest with respect to their therapeutic potential in regenerative medicine. They are attractive for several reasons:

1. Their tissue-appropriate genetic and epigenetic profiles may prevent graft rejection, immune responses and may optimize gene expression.
2. They may be appropriate for autologous transplantation in certain disease states, e.g., AMD, where peripheral MG autografts might be use in a similar fashion to RPE autografts (MacLaren et al., 2007), or they could be used to close recalcitrant macular holes (Yamada et al., 2020).
3. Although currently limited by their proliferative ability, compared to ESCs and iPSCs, they pose minimal ethical and safety concerns (Wang et al., 2020).
4. Endogenous tissue regeneration may be possible through reprogramming (see section “Reprogramming Endogenous Müller Glia for Regenerative Medicine”).

## Müller Glia for the Treatment of Retinal Degeneration

The stem-cell therapeutic potential of MG to treat retinal diseases appears attractive given their well-recognized neurotrophic roles and potential to exhibit a stem/progenitor cell phenotype. However, their stem-cell role(s) *in vivo* in higher vertebrates, including humans, is poorly understood. Over the past decades, protocols have been established to enable the robust culture of MGs *in vitro* via isolation from the retina of adult mammals, including mice, rats, pigs (Pereiro et al., 2020), and even humans (Limb et al., 2002). Notably, in 2002, a spontaneously immortalized human MG (hMG) cell line was isolated from the cadaveric retina and characterized by Limb et al. (2002). Subsequent studies report that most, but not all, cadaveric or surgical retina could give rise to immortalized proliferation (Lawrence et al., 2007; Giannelli et al., 2011), which is perhaps unsurprising given the diversity of genetic makeup and retinal microenvironment amongst human donors, as well as topographical variations in the retinal loci from which samples have been obtained. MG derived both from cadavers (Lawrence et al., 2007) and living donors (Giannelli et al., 2011; Too et al., 2021) have been shown to express stem/progenitor-cell protein markers, such as Sox2, Pax6, and Chx10. Furthermore, they can be induced by various cocktails of growth/differentiation factors for differentiation into post-mitotic retinal cells, such as rod- (Giannelli et al., 2011; Jayaram et al., 2014) and RGC-precursors (Singhal et al., 2012). Recently, MG have been found to release a considerable number of extracellular vesicles, which could potentially be harvested for therapeutic applications (Eastlake et al., 2021).

To further understand the therapeutic potential of MG stem/progenitor cells, researchers performed retinal grafting with the immortalized hMG into the subretinal space of Royal College of Surgeons (RCS) rats (a well-known model of autosomal recessive rod-cone dystrophy) and normal neonatal Lister hooded rats (Lawrence et al., 2007). They observed integration of transplanted cells into different retinal layers, where the cells express markers of retinal neurones resident in the corresponding layers (Lawrence et al., 2007). Moreover, integration and survival of MG can be better achieved in normal neonatal rats compared to dystrophic RCS rats, indicating the environment-dependent efficiency of cell integration (Lawrence et al., 2007). Concurrently, Giannelli et al. (2011) explored the therapeutic potential of differentiated hMG, which were primed for rod photoreceptor commitment by co-culture with PA6 cells, basic fibroblast growth factor withdrawal and taurine supplementation. Following subretinal injection into neonatal immunodeficient mice, these hMG integrated into the outer nuclear layer, where they displayed rod morphology, but lacked outer segments (Giannelli et al., 2011). A subsequent study by Jayaram et al. (2014) investigated functional rescue following subretinal transplantation of hMG and hMG-derived rod precursors into 3-week-old rats with a P23H-1 heterozygous rhodopsin mutation (a murine model of autosomal dominant rod-cone dystrophy). Consistent with previous findings, transplanted hMG were shown to integrate across all retinal layers 4-weeks postoperatively, while differentiated precursors were mainly found in the host outer nuclear layer limited at the injection site. Although the latter lacked mature outer segments, they expressed synaptophysin, thus indicating synaptic connectivity (Jayaram et al., 2014). Dystrophic animal eyes treated with differentiated hMG were also found to display significantly greater a-wave amplitudes on electroretinography than those treated with undifferentiated hMG, or control (untreated) eyes, suggesting the restoration of rod function (Jayaram et al., 2014). Apart from hMG-derived photoreceptor precursors, there has also been success in differentiating hMG into RGC precursors, which, despite the lack of cell integration into host retina, were shown to partially rescue rodent RGC function following RGC depletion, suggesting a neurotrophic mechanism of action (Singhal et al., 2012; Becker et al., 2016).

Müller glia derived from the cadaveric human retina possess the advantage of displaying an indefinite proliferative capacity *in vitro* and have a potentially promising outlook as a homologous stem/progenitor-cell source (Limb et al., 2002). However, disadvantages include the potential for disease transmission (e.g., using “uncorrected” autologous grafted cells in patients with early-onset dystrophies/degeneration) and histocompatibility issues in the case of homologous cells. The first challenge can be addressed via homologous transplantation or through genetic correction in patients with known genotypes, e.g., via CRISPR-Cas9 (Burnight et al., 2017; Gallego et al., 2020). In the case of autologous/homologous hMG, these may be derived from surgical retinae (Too et al., 2021) or harvested from retinal organoids derived from human iPSC or homologous ESC lines that comply with regulatory requirements for clinical development (Nakano et al., 2012; Volkner et al., 2016; Eastlake et al., 2019). As with other stem cell derivatives, such as iPSC-derived photoreceptor precursors, the efficacy of host retinal integration of hMG for functional rescue, and the survival of these cells in the host retinal environment requires improvement. It remains unknown as to what extent hMG and their derivatives may exert a therapeutic effect via neuroprotection, cell replacement, or both. However, there is some evidence of integration, with the formation of synapses: this suggests that their beneficial effects are not simply limited to neuroprotection via material transfer or paracrine actions (Jayaram et al., 2014). It also remains to be seen whether MG reprogrammed into iPSC to generate 3D retinal organoids may produce retinal cells that are more suitable for cellular replacement strategies (Slembrouck-Brec et al., 2019) than those generated by 2D direct differentiation from MG stem/progenitor cells. One proposed advantage of the former is that organoids recapitulate normal retinal development, and hence may produce appropriately staged neuronal cells (Gonzalez-Cordero et al., 2013). Given the versatility of MG, their role in regenerative (cellular or acellular) therapy warrants further investigation.

## Reprogramming Endogenous Müller Glia for Regenerative Medicine

Despite consistent reports that adult hMG possess stem-cell characteristics, neurogenesis is not generally believed to occur in the adult retina of higher vertebrates. This has led to the study of the factors preventing endogenous reprogramming of MG for neuronal regeneration, a process that, by contrast, occurs indefinitely in teleost fish throughout life. In Xenopus, chicken and mice, neuronal regeneration is age-dependent, where the neurogenic potential is higher in early life (Fischer and Reh, 2001; Loffler et al., 2015; Langhe et al., 2017). In the rat, more proliferating cells are found in the peripheral retina of RCS rats than their wild-type counterparts, suggesting activation of retinal stem cells by retinal degeneration (Jian et al., 2009). A similar phenomenon has also been reported in humans, where proliferative vitreoretinopathy triggers activation of neurogenic properties in peripheral MG (Johnsen et al., 2012), a process that ultimately leads not to regeneration, but to a repair mechanism termed “gliosis.” Furthermore, activation of retinal progenitors is proposed as one of the mechanisms of macula hole closure in the case of autografts (Yamada et al., 2020) and indeed may play a role in cases of spontaneous closure and closure following “conventional” macular hole surgery (vitrectomy, inner limiting membrane peeling with gas “tamponade”). Nonetheless, hMG mediated gliosis is a double-edged sword, which may confer both neuroprotective and detrimental effects (Bringmann et al., 2009). How can we tip the balance of the gliotic process to favor a regenerative outcome? Since oculogenesis is highly conserved in vertebrate retinae, understanding the molecular mechanisms underlying MG reprogramming in zebrafish (Lahne et al., 2020) has formed the basis for several research programs seeking to unlock the endogenous reparative capacity of mammalian MG. Moreover, mammalian MG, including hMG (section “Müller Glia for the Treatment of Retinal degeneration”), can proliferate and differentiate into various retinal cells *in vitro*; it is therefore strongly believed that MG could be induced endogenously for retinal regeneration.

The process of MG reprogramming for neuronal regeneration as observed in lower vertebrates generally involves four stages:

1. De-differentiation of resident MG into multipotent stem/progenitor cells.
2. Proliferation of MG-derived stem/progenitor cells.
3. Departure of progenitor cells from the cell cycle, and;
4. Induction of differentiation into retinal neurones.

The biological mechanisms underlying MG reprogramming remain unclear. Transcription factor regulation, growth factor production, cell-cell interaction, the immune microenvironment, and epigenetic modifications may all contribute to the future success of MG reprogramming for neuronal regeneration in the mammalian retina following injury (Gao et al., 2021). For instance, forced expression of achaete-scute homolog 1 (ASCL1), a transcription factor critical for MG reprogramming in zebrafish, fails to induce MG phenotype change in the undamaged mouse retina. However, limited retinal regeneration was observed in the damaged murine retina, with more profound regeneration in younger mice (Ueki et al., 2015). Subsequent epigenetic modification by the HDAC inhibitor TSA, together with ASCL1 overexpression and NMDA-induced damage, further enhanced MG reprogramming, resulting in endogenous *trans*-differentiation of a small population of resident MG into amacrine and bipolar cells in adult mice (Jorstad et al., 2017). Further inhibition of STAT signaling results in a two-fold increase in MG-transdifferentiated neurones (Jorstad et al., 2020). ASCL1 may also collaborate with the RNA-binding protein LIN28 to induce MG reprogramming into multipotent progenitors that express markers for photoreceptors, amacrine cells, bipolar cells and RGCs in NMDA-treated adult mouse retina (Elsaeidi et al., 2018). On the other hand, activation of WNT/ß-catenin signaling pharmacologically, or via adenovirus transfection, stimulates limited MG proliferation in the normal adult mammalian retina via the LIN28/Let-7 miRNA-dependent pathway (Yao et al., 2016). WNT/ß-catenin signaling activation, in combination with transcription factors (OTX2, CRX, and NRL, which are essential for rod cell fate differentiation), further promote MG reprogramming into rod photoreceptors, leading to the functional rescue in the double mutant Gnat1^rd17^Gnat2^cpfl3^ rod & cone deficient mice (Yao et al., 2018). Other pathway manipulations, including downregulation of Ptbp1 (Zhou et al., 2020) and hippo signaling (Rueda et al., 2019) have also been shown to stimulate MG *trans*-differentiation and proliferation/differentiation, respectively, into retinal neurones following NMDA injury. With cross-species transcriptomic and epigenomic analysis, Hoang and colleagues further identified important gene regulatory factors, nuclear factor I transcription factors a, b, and x, that suppress neurogenic competence and lead to quiescence of adult mouse MG following injury (Hoang et al., 2020).

Cell-cell fusion is an essential mechanism occurring during development, and cell fusion-mediated MG reprogramming has been shown to rescue damaged retinal structure and function. For instance, N-Methyl-D-aspartate (NMDA)-induced retinal injury mediates fusion of transplanted hematopoietic stem and progenitor cells (HSPCs), together with activation of Wnt/β-catenin signaling, triggering de-differentiation, proliferation, and generation of amacrine cells and RGCs which can achieve functional rescue (Sanges et al., 2013). In mice with photoreceptor degeneration, transplanted HSPCs-MG hybrids are also able to regenerate functional photoreceptors in addition to providing neuroprotection to residual host neurones (Sanges et al., 2016). Cell-cell fusion-mediated MG reprogramming has also been achieved in the absence of exogenous stem/progenitor cells, via the recruitment of bone-marrow cells to NMDA-injured retina by modulating stromal-cell derived factor-1/CXCR4 signaling (Pesaresi et al., 2018). Likewise, “metabolic reprogramming,” a mechanism that underlies metabolic processes that regulate epigenetic changes associated with stem cell fate, has important implications for endogenous retinal regeneration (Ryall et al., 2015). Mitochondrial transfer from transplanted donor cells to host cells has also been observed; this process leads to functional rescue in a mouse model of RGC degeneration (Jiang et al., 2018, 2019). Pharmacological manipulation of the glycolytic pathway and mitochondrial division also affects somatic cell reprogramming into iPSCs (Vazquez-Martin et al., 2012).

Together, these findings suggest that the determinants of MG reprogramming are multifactorial. Since MG reprogramming does not involve the entire MG population, and given that different MG “stemness” has been shown across retinal regions, there may be present different MG subtypes with varying regenerative capacity: this has yet to be explored fully.

## Conclusion and Future Directions

**FIGURE 2:**
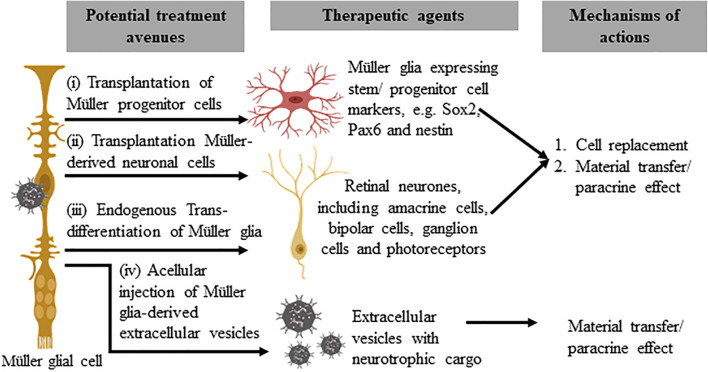
Potential therapeutic approaches that utilize Müller glia and their derivatives. The subpopulation of Müller glia in the mammalian retina that expresses stem/progenitor cell markers may be harvested for direct cellular therapy (i) or differentiated *in vitro* to generate retinal precursor cells before transplantation (ii). These approaches may replace lost retinal cells or confer a neuroprotective effect to remaining host cells, via homologous or autologous transplantation. The third approach involves endogenous reprogramming of Müller glia (iii) to produce retinal cell types that target functional vision restoration of different disease conditions. Acellular therapy with autologous or homologous Müller glia-derived extracellular vesicles (iv), on the other hand, may provide neuroprotective or immunomodulatory effects to treat retinal diseases.

Advances in our understanding of retinal embryogenesis and the stem-cell phenotypes of adult vertebrate retinae suggest MG are a potentially important homologous/autologous/endogenous source of stem/progenitor cells. The therapeutic potential of MG to treat retinal dystrophy and degeneration could be achieved via several approaches: (1) Cellular transplantation of MG progenitor cells, (2) Cellular transplantation of MG-derived retinal cells, (3) Endogenous MG reprogramming, and (4) Acellular therapy with MG-derived extracellular vesicles (Figure 2).

In common with photoreceptor derivatives, or iPSCs derived from different sources, MG obtained from various sources (cadaveric or living donors or ESC/iPSC-derived retinal organoids) may harbor dissimilar regenerative and/or reparative capacities that may only be therapeutically beneficial for certain subsets of degenerative retinal conditions at specific junctures of the disease process. While exogenous cell transplantation offers a potentially pathway-agnostic therapeutic strategy, which may therefore be more suitable for a broader disease spectrum, endogenous reprogramming of MG is appealing because it may ultimately be less invasive and implemented through more straightforward protocols. However, this approach would be unsuitable for certain disease states, e.g., in those with early-onset inherited retinal degeneration, as recapitulation of retinogenesis in such cases is anticipated to reproduce the genotype and phenotype. Despite its seemingly great promise, the extent of endogenous MG reprogramming required for meaningful functional rescue – without sacrificing the structural integrity and homeostasis of the native retina – remains unclear. Given the plethora of roles of MG in governing retinal disease and health, further research is warranted to identify MG and MG-derived neuronal cells or extracellular vesicles that are appropriately staged for optimal functional rescue/restoration.

## Author Contributions

LKT wrote the manuscript. MPS critically revised the manuscript. Both authors contributed to the article and approved the submitted version.

## Conflict of Interest

The authors declare that the research was conducted in the absence of any commercial or financial relationships that could be construed as a potential conflict of interest.

## Publisher’s Note

All claims expressed in this article are solely those of the authors and do not necessarily represent those of their affiliated organizations, or those of the publisher, the editors and the reviewers. Any product that may be evaluated in this article, or claim that may be made by its manufacturer, is not guaranteed or endorsed by the publisher.
